# Genome sequences of human coronavirus NL63 diagnosed in southern France

**DOI:** 10.1128/mra.00841-25

**Published:** 2025-12-16

**Authors:** Houmadi Hikmat, Céline Boschi, Sarah Aherfi, Aurélie Morand, Bernard La Scola, Philippe Colson

**Affiliations:** 1 IHU Méditerranée Infection, Marseille, France; 2 Microbes Evolution Phylogeny and Infections (MEPHI), Aix-Marseille University (AMU), Marseille, France; 3 Assistance Publique-Hôpitaux de Marseille (AP-HM), Marseille, France; 4 Service de Pédiatrie générale, Hôpital Timone, AP-HM, Marseille, France; 5 Service d'accueil des Urgences Pédiatriques, Hôpital Nord, AP-HM, Marseille, France; DOE Joint Genome Institute, Berkeley, California, USA

**Keywords:** coronavirus, human coronavirus-NL63, genome, phylogenomics, genomic surveillance

## Abstract

We report here 17 human coronavirus NL63 genomes from France. They were obtained from residues of respiratory samples collected from patients for diagnostics in southern France, using an in-house multiplex PCR amplification system followed by next-generation sequencing with Illumina technology. Sixteen genomes belong to subgenotype C2 and one to subgenotype B1.

## ANNOUNCEMENT

Human coronavirus NL63 (HCoV-NL63), discovered in 2003 (1), belongs to the genus *Alphacoronavirus* (2) and likely has a zoonotic origin from bats (3). It has a single-stranded positive-sense RNA genome of ≈28 kilobases that encodes 16, 4, and 1 nonstructural, structural (spike, envelope, matrix, and nucleocapsid), and accessory proteins, respectively (4). Its cell receptor is angiotensin-converting enzyme 2 (5). HCoV-NL63 is ubiquitous worldwide (6, 7), causing respiratory tract infections, mostly in children, usually mild but potentially severe and life-threatening (8–12). A total of 345 genomes (>90% coverage of KY073745.1) were released in GenBank (https://www.ncbi.nlm.nih.gov/genbank/) as of 17 July 2025, obtained from patients sampled between 1983 and 2025, in 24% of the cases since 2020, mostly from the USA (31%), UK (28%), and China (21%). Three genotypes A–C and eight subgenotypes were delineated (12–14).

Here, HCoV-NL63 genomes were obtained from residues of HCoV-NL63 RNA-positive respiratory samples collected in 2021 from patients for routine diagnostic by multiplex qPCR (15). RNA was extracted with the MagMAX Viral/Pathogen Nucleic Acid Isolation kit on a KingFisher Flex system (Thermo Fisher Scientific). Thirty-five PCR primer pairs (Table 1) were designed with GEMI (16) and then used to pre-amplify HCoV-NL63 genomes with the SuperScript III One-Step RT-PCR kit with Platinum Taq DNA polymerase (Thermo Fisher Scientific), as previously described (15). Next-generation sequencing was performed as previously described (15) using Illumina technology and the COVIDSeq protocol (Illumina Inc.), with replacement of ARTIC COVID-19 primers with primers designed here. Library preparation and sequencing of 2 × 250 paired-end reads on a MiSeq instrument (Illumina Inc.) were performed following the manufacturer’s instructions. There were, on average, 7,353,541 ± 5,020,276 reads generated per sample (range, 264,636–17,879,700). The Genome Detective web application (https://www.genomedetective.com/) (17) performed a trimming and quality control of reads, then the assembly, annotation, and taxonomic classification of viral genomes with default settings. Phylogeny was performed using IQ-TREE 2 (18).

**TABLE 1:** In-house designed PCR primer pairs used for HCoV-NL63 genome pre-amplification

Primer name	Sequence (5′−3′ orientation)	Pool	Primer concentration in pool (µM)
NL63_F1	ARGTTTGTAAACTGGTTAGGC	1	10
NL63_R1	ACACGCCATAAAGACACACC		10
NL63_F2	ACTGGTGTGTCTTTATGGCG	2	10
NL63_R2	GAAGTAAGTGTAAGCTTAAGAG		10
NL63_F3	AGTATTGATGATGGCCRTTTAT	1	10
NL63_R3	CTGTACAAACACACCAGTAC		10
NL63_F4	GGTACTGGTGTGTTTGTACAG	2	15
NL63_R4	AGAAACACAGTTTAYAAGGTGT		15
NL63_F5	TTCACACCTTRTAAACTGTGTTTC	1	10
NL63_R5	ATCCTTACACCAAAAATACCAC		10
NL63_F6	AATGGTATTCCACTTATGCCTC	2	12.5
NL63_R6	CTGCCTGTTGCAAAATACCAT		12.5
NL63_F7	AATGGTATTTTGCAACAGGCA	1	12.5
NL63_R7	TTCTTAAAACAGTCGTTAACATCC		12.5
NL63_F8	AATCTTGGTCGTTGTGTGCG	2	12.5
NL63_R8	CATTAGATGYTACACAACCAC		12.5
NL63_F9	TGTCTACTYTAGCTGTTACAGC	1	12.5
NL63_R9	TAATATAGGATCRCGAATGTGC		12.5
NL63_F10	TGGAAGCACATTCGYGATCC	2	10
NL63_R10	ATAAYATATGCAGGAGCTGTG		10
NL63_F11	GGCCTGGTAAYACTTTTATTAA	1	10
NL63_R11	ACCAACAACTATAGGACATTTATC		10
NL63_F12ALT	GGCATGAGGCTAAGTTTGGT	2	10
NL63_R12ALT	AGCCTCACTAGCACTACCA		10
NL63_F13	GGTACTTTTGAGAGTGCTGC	1	10
NL63_R13	CAAGCTCCATTTATAAAAGAAC		10
NL63_F14	GGAGCTTGTGGTTCTCCTG	2	10
NL63_R14	AGGTGTAAGTATMACAGGGTT		10
NL63_F15	TCTTCACAATGTTTTGGGCAG	1	10
NL63_R15	TAAATTCAGCAGCACTMACCT		10
NL63_F16	GTTTGTACTTATTGGGGCATT	2	10
NL63_R16	CAAATGATGAAGCAACACTCT		10
NL63_F17	GATTTTGGTCTTGATGGCCTT	1	10
NL63_R17	CAAATACTTCACTTGAGGACC		10
NL63_F18	GTGGGAGTATGAGGGTGG	2	12.5
NL63_R18	ATTACCATAAATGACTCTGCC		12.5
NL63_F19	CTGGTGCTTTGGCTGAGC	1	10
NL63_R19	AACCCATAAYAGGCATCATATA		10
NL63_F20	CCTAATATGGGTGTTCCCTG	2	10
NL63_R20	ACACCATCRATTAAAGTACGC		10
NL63_F21	ACACGCAATGCYACTGTTGT	1	8.75
NL63_R21	CGGTGTTATAGCCAAAATAAAC		8.75
NL63_F22	TTGTGGTGATTGTYTGCGTAA	2	12.5
NL63_R22	GTAATCATTACTCTAGGTGCA		12.5
NL63_F23	GGTGAYCCACAACAACTTCC	1	8.75
NL63_R23	CACAAAATATACCYTTCTTAGC		8.75
NL63_F24	ACTTCTGACACTGCACATGC	2	8.75
NL63_R24	GTCCCTGGACATTACGCC		8.75
NL63_F25_ALT	TCATAGAAACGAGCATGATGC	1	10
NL63_R25_ALT	GTCAAAACAAACACAAACATCC		10
NL63_F26	ATTCAGGGTTCGTATGAGCG	2	12.5
NL63_R26	CTTCAAGRTAAACAGTAGCAC		12.5
NL63_F27	TCCACARTTGCAGTCTGCTG	1	15
NL63_R27	TTCACCMGATATAGTTATRC		15
NL63_F28	CAGAACAGTTAGGTGCGCC	2	15
NL63_R28	CACAAAAGTGCTAACATGAGT		15
NL63_F29	AACACYACTCATGTTAGCACT	1	10
NL63_R29	CTCACGAATACCAGARACAG		10
NL63_F30	ATACTATTGTTGGTGCTTTGTAT	2	12.5
NL63_R30	AGTACCCAAACCAGATGTAACA		12.5
NL63_F31_ALT	TGCCTTACGACTTAGTGCTC	1	15
NL63_R31_ALT	ATTAACATCAACGTAGTCAGGT		15
NL63_F32	CTGTCATACCTGACTACGTT	2	15
NL63_R32	ATCGAAGGAACATCTTCGTATA		15
NL63_F33	GCACCTGTTCCAGCTGAAG	1	8.75
NL63_R33	CCCCCTRCGCATACGCC		8.75
NL63_F34	GGCGTATGCGYAGGGGG	2	10
NL63_R34	GAGTCTCGTGAGTTGTTACG		10
NL63_F35	TCTGTTGTTGAGTTYGAGGAT	1	15
NL63_R35	TTACACTTTACTATCACTGGC		15

**Fig 1:**
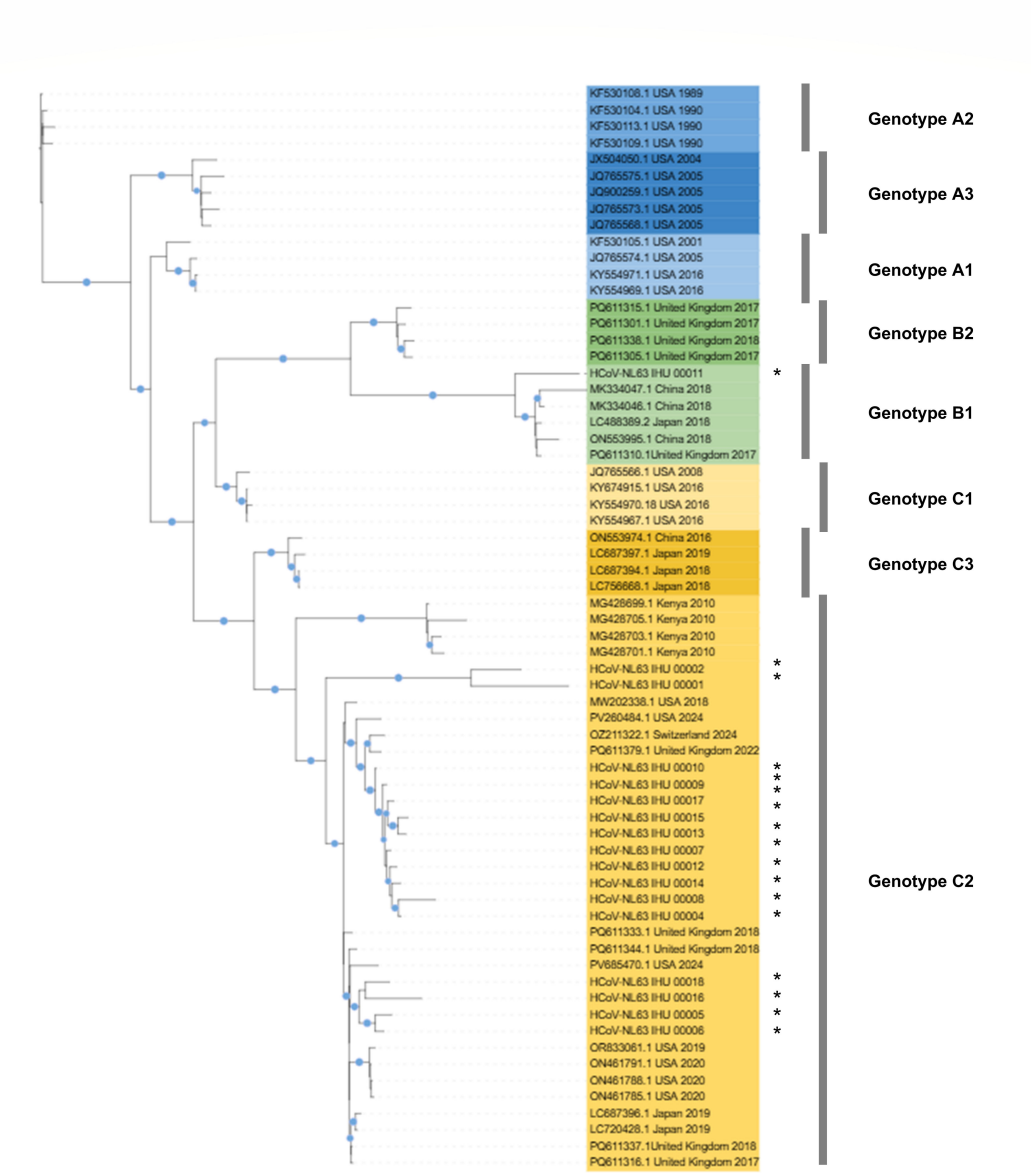
Phylogeny reconstruction based on HCoV-NL63 genomes recovered in the present study or available from GenBank. Sequence alignment was performed using the MAFFT v.7 web application (https://mafft.cbrc.jp/alignment/server/index.html) with automatic parameter optimization and multithreading to improve speed and accuracy. Phylogeny reconstruction was performed using IQ-TREE 2 (18) using the maximum likelihood method according to the TN + FO + I model, with branch support assessed using 1,000 bootstrap replicates. The phylogenetic tree was visualized using iTOL (https://itol.embl.de). HCoV-NL63 genomes incorporated in the phylogenetic analysis included genomes from the different previously delineated lineages or sublineages and the five best BLAST hits in GenBank for each of the genomes obtained here. Blue circles indicate bootstrap values >80%. Genomes obtained here are indicated by a black star.

Seventeen HCoV-NL63 genomes, 27,322–27,465 nucleotides long, were obtained. Mean coverage relative to genome NC_005831.2 dating back to 2003 was 98.0% (range, 90.0–99.9%). Coverage <100% was due to amplification defects of some regions, mostly 5′ and 3′ genome ends. Phylogeny identified subgenotypes C2 and B1 in 16 and one case, respectively (Fig. 1). Subgenotype C2 genomes exhibited a similarity of 98.3% on average between each other and of 90.5% with the subgenotype B1 genome. The closest relatives according to BLAST searches into GenBank and to phylogeny were obtained from the USA, Japan, UK, China, and Switzerland between 2017–2024; mean similarity was 97.3% with the best hits. Substitution I507L, located in the spike receptor binding domain and suspected to promote viral entry into host cells (12), was present in all subgenotype C2 genomes but absent in the subgenotype B1 genome.

In summary, the HCoV-NL63 genomes provided here from France account for around a fifth of those available worldwide since 2020. They evidence that at least subgenotypes C2 and B1 circulated in our geographical area. Prior evidence of expanding diversity and of frequent recombinations (19, 20) warrants intensifying HCoV-NL63 genome sequencing retrospectively and prospectively to get a more detailed picture of the epidemiology, diversity, and evolution of this virus.

## Data Availability

The HCoV-NL63 genomes analyzed here have been submitted to the GenBank sequence database (https://www.ncbi.nlm.nih.gov/genbank/) under accession no. PX111341 to PX111357. Next-generation sequencing raw data have been submitted to the NCBI Sequence Read Archive (SRA) (https://www.ncbi.nlm.nih.gov/sra) under BioProject accession no. PRJNA1308010 and SRA no. SRX30150765 to SRX30150781.
